# Spatially optimized 125I brachytherapy: a novel immunomodulatory approach based on spatially fractionated radiation therapy principles

**DOI:** 10.3389/fonc.2025.1693574

**Published:** 2025-12-05

**Authors:** Qiyu Sun, Yanbo Hu, Zhiqian Sun, Lulu Du, Rui Huang, Lichao Wang, Zhenzhen Cui, Jiaxin Yang, Xiaowen Ma, Min Li

**Affiliations:** 1 The 960th Hospital of People’s Liberation Army (PLA), Jinan, China; 2 Department of Nuclear Medicine, The Postgraduate Training Base of Jinzhou Medical University The 960th Hospital of People's Liberation Army (PLA), Jinan, China; 3 Shandong Second Medical University, Weifang, China

**Keywords:** 125I brachytherapy, immunomodulatory, SFRT principles, ICD, tumors

## Abstract

Conventional ¹²^5^I brachytherapy, emphasizing uniform dose coverage, may limit its potential to activate anti-tumor immunity. We propose integrating the principles of Spatially Fractionated Radiotherapy (SFRT) into ¹²^5^I brachytherapy. Through a heterogeneous dose design featuring “co-existing high- and low-dose regions”, this approach aims to synergize immunogenic cell death (ICD) with the preservation of immune cell function, thereby transforming the tumor into an “*in situ* vaccine”. The inherent characteristics of ¹²^5^I sources – namely, the steep dose gradient of their low-energy gamma rays and continuous low-dose-rate delivery – provide unique advantages for the precise construction of distinct regions: a high-dose zone (to induce ICD) and a low-dose zone (to support immune cell survival).We have established a novel dose evaluation framework (incorporating metrics such as HDRI [High-Dose Region Index], LDRI [Low-Dose Region Index], DGUI [Dose Gradient Utilization Index], and SHS [Spatial Heterogeneity Score]) to replace traditional homogeneous indices like D90/V100. Furthermore, we present optimization strategies for parameters including source spacing and source activity. Finally, we systematically outline an integrated preclinical-to-clinical research pathway for combination with immunotherapy. This lays the theoretical and experimental foundation for developing tumor-type-specific, personalized treatment protocols based on the “¹²^5^I-SFRT + Immunotherapy” approach.

## 1 Introduction to SFRT and iodine-125 brachytherapy

Over the past decades, radiation therapy has been increasingly recognized for its potential to stimulate antitumor immune responses beyond its direct cytotoxic effects. Emerging evidence suggests that radiation can convert tumors into “*in situ* vaccines” by inducing immunogenic cell death, releasing tumor antigens, and remodeling the tumor microenvironment (1, 2). Among various radiation delivery approaches, spatially fractionated radiation therapy (SFRT) has demonstrated unique advantages in immune modulation through its distinctive non-uniform dose distribution pattern (3). SFRT delivers alternating regions of high and low doses within the target volume, creating a spatial dose modulation that differs fundamentally from conventional uniform dose delivery (4, 5). The biological effectiveness of this approach stems from the distinct immunological responses triggered in different dose regions. High-dose regions, typically receiving 2–3 times the conventional dose, induce immunogenic cell death (ICD) through the release of damage-associated molecular patterns (DAMPs), including HMGB1, calreticulin, and ATP (6). These molecular signals activate dendritic cells and enhance their ability to present tumor antigens to T cells. Meanwhile, low-dose regions, receiving approximately 20-30% of the prescription dose, create an environment conducive to immune cell survival and function (7, 8). This spatial arrangement has demonstrated remarkable immunological benefits, as evidenced by enhanced CD8+ and CD4+ T cell infiltration, improved dendritic cell recruitment and function, and favorable reshaping of the tumor microenvironment through the modulation of tumor-associated macrophages from an immunosuppressive M2 to an immunostimulatory M1 phenotype (5). The biological rationale behind SFRT’s effectiveness lies in this synergistic interplay between high-dose regions that trigger immunogenic cell death and low-dose regions that preserve immune cell functionality, ultimately fostering a more effective anti-tumor immune response. Iodine-125 (125I) brachytherapy, as a well-established treatment modality, has been widely used in clinical practice due to its unique physical properties. The low-energy gamma emissions and prolonged half-life of 125I sources create sustained radiation exposure in the tumor vicinity. The conventional approach to 125I brachytherapy planning primarily focuses on achieving uniform dose coverage, adhering to standardized dosimetric parameters such as D90 and V100 (9, 10). This emphasis on dose uniformity may paradoxically limit the treatment’s immunological potential, as emerging evidence suggests that heterogeneous dose distributions could better activate anti-tumor immune responses. This paper proposes a novel hypothesis that integrating SFRT principles into 125I brachytherapy planning could enhance its immunomodulatory effects. By optimizing the spatial distribution of 125I sources, we could potentially create controlled patterns of high and low dose regions that mirror the immunological benefits observed with external beam SFRT. The continuous low-dose-rate radiation characteristic of 125I sources may offer unique advantages in sustaining immune responses compared to the acute exposure of external beam radiation (11).

## 2 Theoretical foundation

The potential advantages of integrating SFRT principles into 125I brachytherapy are grounded in both the established immunological mechanisms of SFRT and the unique physical properties of 125I sources. Understanding these fundamental aspects provides the theoretical basis for optimizing brachytherapy spatial distribution to enhance immune responses.

### 2.1 Immunomodulatory mechanisms of SFRT

SFRT’s ability to enhance anti-tumor immunity has been well-documented through extensive preclinical studies (12, 13). The spatial distribution of radiation doses creates distinct microenvironmental zones that work in concert to stimulate immune responses. In high-dose regions, radiation-induced stress triggers the exposure of calreticulin on the cell surface and the release of ATP and HMGB1, marking the initiation of immunogenic cell death. This process converts dying tumor cells into effective *in situ* vaccines, providing a rich source of tumor antigens for immune recognition. The preservation of immune cell functionality in low-dose regions represents another crucial aspect of SFRT’s effectiveness (13). These areas maintain viable immune cells while supporting their activation and function. Studies have demonstrated that dendritic cells in low-dose regions exhibit enhanced antigen-presenting capabilities, facilitating more effective T cell priming. Furthermore, the dose gradient between high and low-dose regions creates chemokine gradients that guide immune cell trafficking, promoting the infiltration of cytotoxic T lymphocytes and natural killer cells into the tumor microenvironment.

### 2.2 Advantages of 125I brachytherapy

125I sources possess distinct physical characteristics that make them particularly suitable for implementing SFRT principles. The low-energy gamma emissions (average 28 keV) naturally create steep dose gradients around each source, with dose rates decreasing rapidly with distance (14). This inherent property allows for precise control over the spatial distribution of radiation doses through strategic source placement (5). Moreover, the 59.4-day half-life of 125I provides sustained low-dose-rate radiation, potentially offering advantages over the acute exposure typically delivered in external beam SFRT. The continuous nature of 125I irradiation may be particularly beneficial for immune response modulation. Rather than delivering radiation in discrete fractions, 125I sources provide uninterrupted low-dose-rate exposure that could maintain persistent immune stimulation. “The sustained low-dose-rate (LDR) exposure from ^125^I seeds (typically 0.05–0.2 Gy/h) creates a dynamic equilibrium: While radiation-induced cell death occurs continuously, the rate of antigen/DAMP release remains within the processing capacity of dendritic cells. This ‘trickle effect’ avoids exhausting immune responses, while chronic stress selectively eliminates radiosensitive Treg cells. Concurrently, effector T cells upregulate DNA repair pathways (e.g., ATM/Chk2), enhancing their survival under LDR. To prevent cumulative damage, we emphasize strict dose-rate control (<0.3 Gy/h) and combination with TLR9 agonists or CD47 blockade, which convert antigen persistence into durable immune activation” (15). This sustained radiation delivery might better support the development of immunological memory and potentially enhance the durability of anti-tumor responses.

### 2.3 Synergistic potential

The integration of SFRT principles with 125I brachytherapy presents several theoretical advantages for immune modulation: First, the ability to precisely position 125I sources allows for the creation of controlled patterns of high and low dose regions within the tumor volume. By optimizing source placement and activity, it becomes possible to generate immunologically favorable dose distributions that mirror the beneficial effects observed with external beam SFRT (16). Second, the sustained low-dose-rate radiation characteristic of 125I may provide more gradual and persistent immune stimulation compared to the acute exposure of external beam radiation. This could potentially lead to more effective priming of anti-tumor immune responses and better maintenance of immune cell function (17). Third, the local nature of brachytherapy, combined with the short range of 125I radiation, may help preserve critical immune cell populations in the surrounding normal tissues, supporting their participation in the anti-tumor response.

### 2.4 Optimization strategy

The successful implementation of SFRT principles in 125I brachytherapy requires careful optimization of source distribution parameters to achieve the desired immunological effects. This optimization strategy encompasses both spatial arrangement and dosimetric considerations, with the ultimate goal of creating an environment conducive to enhanced immune responses.

### 2.5 Spatial distribution parameters

The spatial arrangement of 125I sources represents a critical factor in achieving the immunomodulatory benefits associated with SFRT (18, 19). Traditional brachytherapy planning typically aims for uniform dose coverage, but implementing SFRT principles requires a paradigm shift toward controlled dose heterogeneity. The optimization of source distribution should consider several key parameters: Source Spacing The distance between adjacent 125I sources significantly influences the formation of high and low dose regions. Based on the physical properties of 125I, including its gamma emission characteristics and dose fall-off pattern, optimal spacing should be determined to create distinct immunologically active zones. The high-dose regions surrounding each source should be sufficient to trigger immunogenic cell death, while maintaining adequate low-dose regions to preserve immune cell function. Activity Distribution Beyond physical spacing, the activity of individual sources can be modulated to fine-tune the dose distribution pattern. This approach allows for greater flexibility in creating the desired dose gradients. Higher activity sources can be strategically placed to generate robust immunogenic cell death in specific regions, while lower activity sources can help maintain immune-preserving zones (17).

## 3 Dosimetric considerations

### 3.1 Limitations of current TPS evaluation methods

Traditional treatment planning systems for 125I brachytherapy rely on standardized dose evaluation parameters such as D90, V100, and dose homogeneity index (DHI) (20, 21). While these metrics effectively assess uniform dose coverage, they are insufficient for evaluating SFRT-style dose distributions where controlled heterogeneity is desired (22). The current evaluation framework lacks the capability to quantify and optimize the spatial characteristics of high and low dose regions that are fundamental to SFRT. Limitations of Current TPS Evaluation Methods.

### 3.2 Proposed new evaluation framework

To effectively implement SFRT principles in 125I brachytherapy planning, we propose a novel dose evaluation framework that focuses on characterizing dose heterogeneity patterns: Spatial Dose Distribution Analysis. Development of sub-volume dose gradient indices to quantify the transition between high and low dose regions. Implementation of three-dimensional dose clustering analysis to evaluate the size and distribution of high and low dose regions. Creation of metrics to assess the connectivity and interfaces between different dose levels. We propose the utilization of grid-based spatially fractionated external beam radiation as a control arm in such studies.

### 3.3 Novel dosimetric parameters

A new set of evaluation parameters should be established to characterize SFRT-specific dose distributions: High-Dose Region Index (HDRI): Quantifying the volume and distribution of regions receiving >150% of prescription dose. Low-Dose Region Index (LDRI): Assessing the volume and distribution of regions receiving 20-50% of prescription dose. Dose Gradient Uniformity Index (DGUI): Evaluating the consistency of dose gradients between high and low dose regions. Spatial Heterogeneity Score (SHS): Measuring the overall non-uniformity pattern of dose distribution.

## 4 Integration with immunotherapy

The successful integration of 125I brachytherapy-based SFRT with immunotherapy requires systematic investigation through both preclinical and clinical studies to establish evidence-based combination strategies. This research pathway must begin with fundamental preclinical investigations to understand the complex interplay between spatially optimized radiation delivery and immune system activation (12). In the preclinical phase, animal models will serve as essential platforms for investigating both spatial and temporal aspects of the combination therapy. Through careful examination of different tumor models, we can determine how varying patterns of high and low dose distributions affect immune responses (23). This research will establish critical parameters including the optimal ratio of high to low dose regions for maximizing immunogenic cell death, threshold doses required in high-dose regions to effectively trigger ICD, and permissible dose ranges in low-dose regions that maintain immune cell function (24). Additionally, these studies will reveal how dose gradients influence immune cell trafficking and functionality within the tumor microenvironment (25). The temporal aspects of combining immunotherapy with 125I SFRT require equally rigorous investigation. Studies must examine the kinetics of radiation-induced immune responses following 125I implantation and determine the optimal timing for introducing different immunotherapy agents. Understanding the duration of radiation-enhanced immune responses will guide decisions about potential multiple cycles of immunotherapy administration. Different cancer types present unique immunological characteristics that necessitate distinct optimization strategies (26). Research must examine how optimized 125I SFRT affects poorly immunogenic “cold” tumors compared to highly immunogenic “hot” tumors, as this understanding will inform whether different dose distribution patterns are needed based on the baseline immune status of the tumor (27). Additionally, the influence of different tissue environments on combination therapy efficacy must be evaluated, particularly for cancers in immunologically privileged sites or those with unique stromal compositions (28). Following successful preclinical optimization, clinical validation should proceed through a carefully structured series of trials. Initial Phase I studies will evaluate the safety profile of various spatial dose distributions and the tolerability of different immunotherapy integration schedules. These studies will also provide preliminary evidence of immune activation and help identify potential biomarkers for treatment response. Subsequent Phase II studies will focus on cancer-specific protocols optimized based on preclinical findings, examining the correlation between dose distribution patterns and treatment outcomes while validating optimal timing sequences for specific immunotherapy agents.

The final stage of validation will involve large-scale randomized controlled trials comparing optimized combination protocols against standard treatments. These comprehensive studies will validate cancer-specific approaches and establish the definitive evidence needed for clinical adoption. The results will guide the development of standardized treatment guidelines while maintaining flexibility for personalized adjustments based on individual patient characteristics. This systematic approach to investigating the integration of 125I SFRT and immunotherapy will provide the robust evidence needed to establish optimal treatment protocols for different cancer types. Through careful attention to both spatial and temporal aspects of the combination therapy, we can develop personalized strategies that maximize therapeutic benefit while maintaining safety. The findings from this research pathway will ultimately transform our approach to cancer treatment by leveraging the synergistic potential of spatially optimized radiation delivery and immunotherapy.

**Figure 1:**
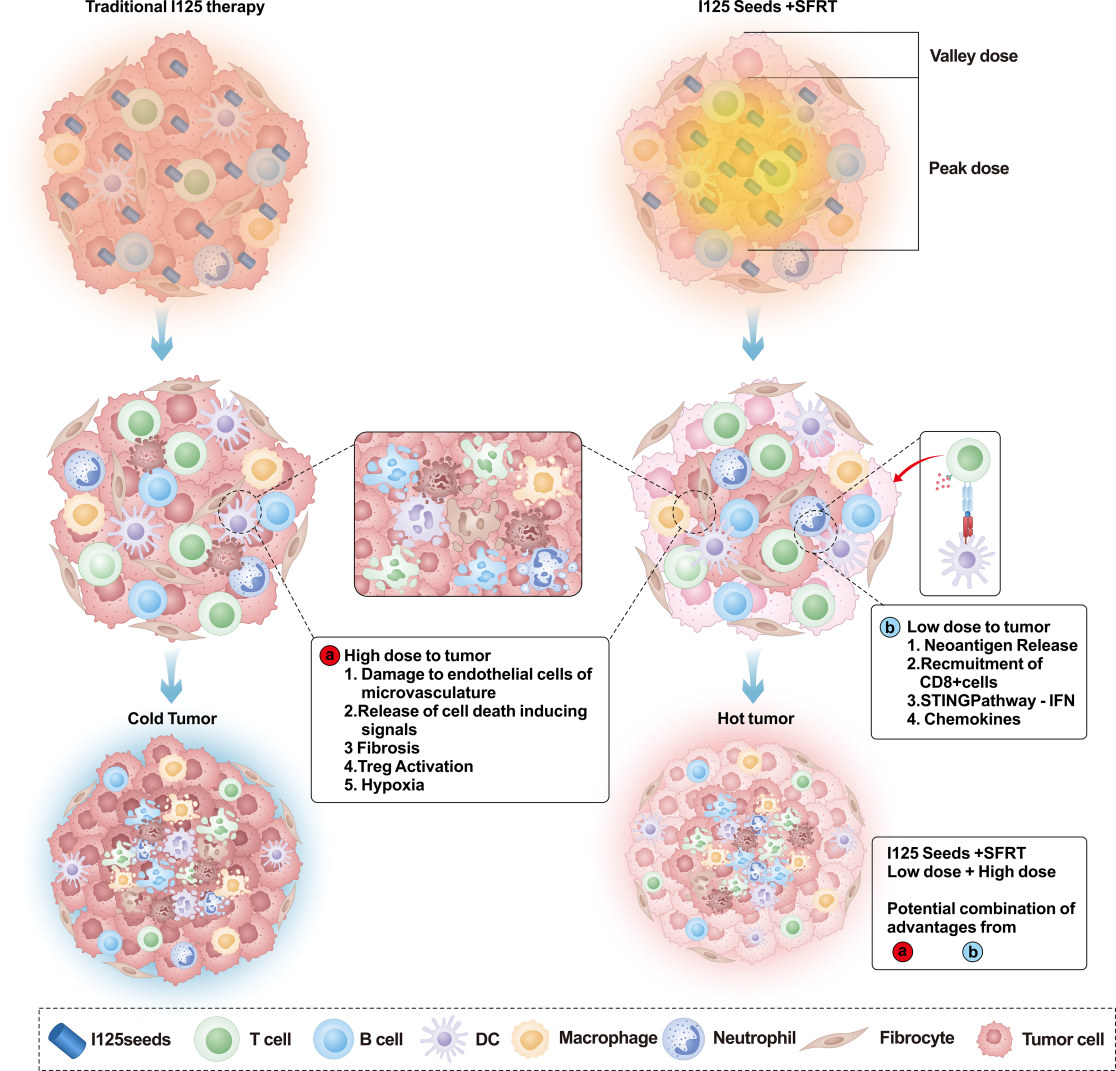
Schematic overview comparing the local effects induced by conventional Iodine-125 (¹²^5^I) brachytherapy and combined ¹²^5^I brachytherapy with Spatially Fractionated Radiotherapy (SFRT) principles on a local tumor. In conventional ¹²^5^I brachytherapy of the tumor, a uniformly high radiation dose is delivered. This high-dose exposure exerts three main effects on the tumor: Endothelial cell death and vascular damage; Induction of CD8^+^ T cells; Release of cell death signals. Current evidence suggests that high-dose radiation can also induce immunosuppression at the tumor periphery. In combined ¹²^5^I brachytherapy + SFRT treatment, the radiation dose is spatially optimized: The tumor center (indicated in the figure) receives a high-dose region, eliciting the same three effects described above for conventional treatment. The tumor periphery receives a low-dose region. This low-dose radiation induces immunomodulatory effects through several pathways: Release of neoantigens; Recruitment of CD8^+^ T cells towards the tumor periphery; Activation of Interferon (IFN) pathway genes.

Therefore, this strategy would potentially combine the inherent benefits of distinct dose regions while avoiding the potential pitfall of high-dose radiation inducing immunosuppressive effects throughout the entire tumor (**Figure 1**).

## 5 Conclusion

The integration of SFRT principles into 125I brachytherapy planning represents a transformative approach that could significantly enhance cancer treatment outcomes (5). This novel strategy leverages the unique physical properties of 125I sources and the established immunological benefits of spatial dose modulation to create a more effective therapeutic paradigm (17). By carefully optimizing source distribution to generate controlled patterns of high and low dose regions, this approach aims to enhance both local tumor control and systemic anti-tumor immunity (12).The framework presented in this paper establishes a comprehensive roadmap for development and implementation, encompassing theoretical foundations, technical optimization strategies, and clinical validation pathways. The proposed dosimetric evaluation system moves beyond traditional metrics to better characterize and optimize the spatial heterogeneity essential for immune activation. The integration with immunotherapy, guided by systematic preclinical studies and carefully designed clinical trials, offers the potential for powerful combination treatments tailored to specific cancer types and individual patient characteristics (29). While significant challenges remain in terms of technical implementation, biological validation, and clinical translation, the potential benefits warrant continued investment in this field. Success in this endeavor could fundamentally change our approach to brachytherapy, transitioning from a focus on uniform dose coverage to biologically-optimized treatment planning that enhances immune system engagement. As our understanding of radiation-immune interactions continues to evolve, this approach may become an integral component of modern cancer therapy, offering new hope for improved patient outcomes through enhanced local and systemic anti-tumor responses. This paradigm shift in 125I brachytherapy planning not only addresses current limitations but also opens new avenues for personalized cancer treatment. By combining the precision of brachytherapy with the power of immune modulation, this strategy represents a promising direction in the ongoing effort to improve cancer treatment outcomes and potentially extend the benefits of local therapy to systemic disease control.

## Data Availability

The original contributions presented in the study are included in the article/**Supplementary Material**. Further inquiries can be directed to the corresponding authors.
