# Supplementary material for: Spatially optimized 125I brachytherapy: a novel immunomodulatory approach based on spatially fractionated radiation therapy principles
Source: Front Oncol. 2025 Dec 5;15:1693574. doi: 10.3389/fonc.2025.1693574 (PMC12714662; doi:10.3389/fonc.2025.1693574)
Supplement: Supplementary file 1 [file DataSheet1.pdf]

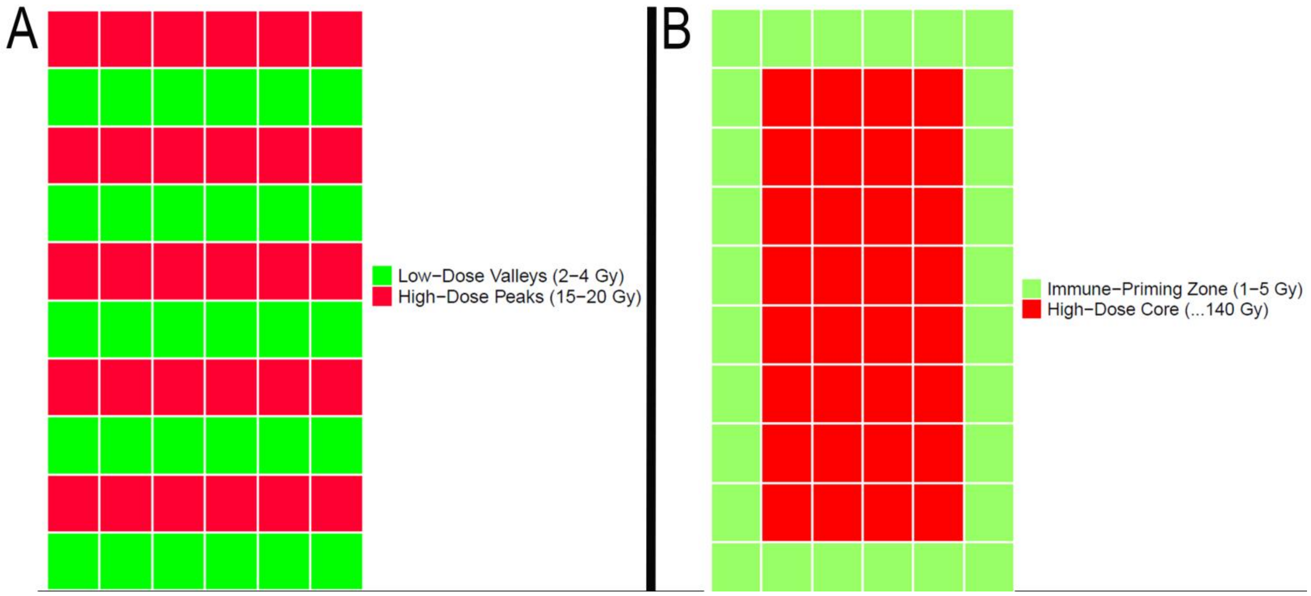

Supplementary Figure 1. 2D Dose Cloud Comparison of SFRT and Brachytherapy.(A) SFRT dose distribution in 5-cm tumor (microbeam spacing: 10 mm).(B) Brachytherapy with ^{125}I seeds (5 mm spacing). Color bar: dose in Gy.
